# Lack of Association of the TP53BP1 Glu353Asp Polymorphism with Risk of Cancer: A Systematic Review and Meta-Analysis

**DOI:** 10.1371/journal.pone.0090931

**Published:** 2014-03-06

**Authors:** Lei Liu, Jinghua Jiao, Yu Wang, Dong Zhang, Jingyang Wu, Desheng Huang

**Affiliations:** 1 Department of Ophthalmology, The First Affiliated Hospital, China Medical University, Shenyang City, Liaoning Province, China; 2 Department of Anesthesiology, Fengtian Hospital, Shenyang Medical College, Shenyang City, Liaoning Province, China; 3 Department of Development and Planning office, China Medical University, Shenyang City, Liaoning Province, China; 4 Department of General Surgery, the Fourth People’s Hospital of Shenyang, Shenyang City, Liaoning Province, China; 5 Department of Epidemiology, School of Public Health, China Medical University, Shenyang City, Liaoning Province, China; University of North Carolina School of Medicine, United States of America

## Abstract

**Objective:**

The TP53BP1 gene may be involved in the development of cancer through disrupting DNA repair. However, studies investigating the relationship between TP53BP1 Glu353Asp (rs560191) polymorphism and cancer yielded contradictory and inconclusive outcomes. In order to realize these ambiguous findings, a meta-analysis was performed to assess the association between the TP53BP1 Glu353Asp (rs560191) polymorphism and susceptibility to cancer.

**Methods:**

We conducted a search of all English reports on studies for the association between the TP53BP1 Asp353Glu (rs560191) polymorphism and susceptibility to cancer using Medline, the Cochrane Library, EMbase, Web of Science, Google (scholar), and all Chinese reports were identified manually and on-line using CBMDisc, Chongqing VIP database, and CNKI database. The strict selection criteria and exclusion criteria were determined, and odds ratios (ORs) with 95% confidence intervals (CIs) were used to assess the strength of associations. The fixed or random effect model was selected based on the heterogeneity test among studies. Publication bias was estimated using funnel plots and Egger’s regression test.

**Results:**

A total of seven studies were included in the meta-analysis including 3,213 cases and 3,849 controls. The results indicated that the Glu353Asp (rs560191) polymorphism in TP53BP1 gene had no association with cancer risk for all genetic models. In the subgroup analysis, the results suggested that Glu353Asp polymorphism was not associated with the risk of cancer according to ethnicity, cancer type, genotyping method, adjusted with control or not, HWE and quality score.

**Conclusions:**

This meta-analysis suggested that the Glu353Asp (rs560191) polymorphism in TP53BP1 gene was not associated with risk of cancer.

## Introduction

It was reported that there were about 12.7 million new cancer cases and 7.6 million cancer deaths through out the world in 2008 [1]. However, the etiology of cancer remains unknown and disease-modifying treatments are limited. In addition, since the involvement of cytokines in cancer was hypothesized, there were many candidate genes approaching in designing a case-control association study of single nucleotide polymorphisms (SNPs) including p53-binding protein 1 (TP53BP1).

TP53BP1 gene has played an important role in both DNA repair and cell cycle control and also mediates the DNA damage checkpoint through cooperation with damage sensors and signal transducers [2]. The TP53BP1 contains two BRCA1 C-terminal (BRCT) domains, which are essential for tumor suppressor functions [3]. The SNPs for TP53BP1 gene may play an important role in the etiology of cancer because of a direct role of TP53BP1 in the cellular response to DNA damage. Previous researches have revealed that no association between TP53BP1 Asp353Glu (rs560191) SNPs and cancer risk [4]–[9], but Kiyohara et al. reported that the Glu/Glu genotype of TP53BP1 Asp353Glu was associated with a decreased risk of lung cancer [10]. So the results of studies concerning association between Asp353Glu (rs560191) polymorphism in TP53BP1 gene and risk of cancer are conflicting.

Considering a single study may lack the power to provide a reliable conclusion, we performed a meta-analysis on these eligible studies to investigate the precise relationship between TP53BP1 Asp353Glu (rs560191) polymorphism and susceptibility to cancer, which would have a much greater possibility of reaching reasonably strong conclusions.

## Methods

### Selection of Eligible Studies

We searched Medline (US National Library of Medicine, Bethesda, MD), Embase, the Cochrane Library, Chinese Biological Medicine, China National Knowledge Infrastructure, Wang Fang Data and Chongqing VIP database (Last search was updated on December 20, 2013) using the terms “p53-binding protein 1 or TP53BP1 or 53BP1”, “Asp353Glu or rs560191 or D353E”, “cancer or tunor or carcinoma” and “polymorphism, variant or mutation”. The selection was done without restriction on language, but we only included published articles written in English or Chinese. We used the PubMed option “Related Articles” for each study to retrieve additional potentially relevant articles. Reference lists were checked and researchers were contacted for additional literatures.

### Selection Criteria

Studies were selected if they met the following criteria: (1) association study with a case-control or cohort design; (2) the study investigated the association between TP53BP1 (rs560191) polymorphism and the risk of cancer; (3) in the case of multiple publications from the same study group, the most complete and recent results were used.

### Exclusion Criteria

The exclusion criteria were defined as: 1) abstracts, reviews and animal studies; 2) useless data reported, genotype number or frequency not included; and 3) study without sufficient data for meta-analysis. If more than one study was published by the same authors using the same case series, only the most recent study or the study with the largest size of samples was included in our meta-analysis.

### Data Extraction

Two reviewers (Lei Liu and Jinghua Jiao) independently scrutinized studies on the associations between TP53BP1 Asp353Glu (rs560191) polymorphism and risk of cancer. When discrepancies were appeared, all investigators were recruited to assess the data. The following information was collected: First author, publication year, location, ethnicity, sample sizes of patients and controls, study design and genotype numbers.

The reviewers developed a quality assessment scale (Table 1), which was modified from previous studies [11]–[13], to evaluate the quality of eligible studies.

**Table 1:** Scale for quality assessment.

Paramete	Score
Source of cases	
Selected from population o rcancer registry	2
Selected from oncology department or cancer institute	1
No description	0
Representativeness of controls	
Population-based	2
Population-hospital mixed	1.5
Hospital-based	1
No description	0
Diagnosis of cancer	
Histological or pathologically confirmed	2
Patient medical record	1
No description	0
Specimens of cases for genotyping	
Peripheral blood or normal tissues	2
Tumor tissues or exfoliated cells	1
No description	0
Quality control of genotyping	
Different genotyping assays confirmed the result	2
Quality control by repeated assay	1
No description	0
Total sample size	
>1000	2
200–1000	1
<200	0

The review and analysis were guided to conduct by the PRISMA statement for preferred reporting of systematic review and meta-analysis [14].

### Statistical Analysis

Odds ratios (ORs) with 95% confidence intervals (CIs) for genotypes and alleles were used to assess the strength of association between TP53BP1 Asp353Glu (rs560191) polymorphism and risk of cancer. The ORs were performed for the allele contrasts, additive genetic model, as well as recessive genetic model and dominant genetic model, respectively. Heterogeneity was examined with *I^2^* statistic interpreted as the proportion of total variation contributed by between-study variation. We also measured the effect of heterogeneity using a quantitative measure, *I^2^* = 100%×(Q−d f)/Q. If there was a statistical difference in terms of heterogeneity (P<0.10, *I^2^>50%*), the random effects model would be used to estimate the pooled ORs [15], [16]. Otherwise, the pooled ORs were estimated by the fixed effects model [17]. Sensitivity analysis was carried out by deleting one single study each time to examine the influence of individual data set on the pooled ORs. The possible publication bias was assessed with funnel plots and Egger’s test. An asymmetric plot suggests a possible publication bias and the P value of Egger’s test less than 0.05 was considered representative of statistically significant publication bias [18]. All statistical tests were performed with RevMan version 5.0 (Review Manager, Copenhagen: The Nordic Cochrane Centre, The Cochrane Collaboration, 2010) and Comprehensive Meta-Analysis software version 2.0 (Biostat, Englewood Cliffs, I.N.J., USA). *P* value of smaller than 0.05 for any test was considered to be statistically significant.

## Results

### Study Inclusion and Characteristics

**Figure 1:**
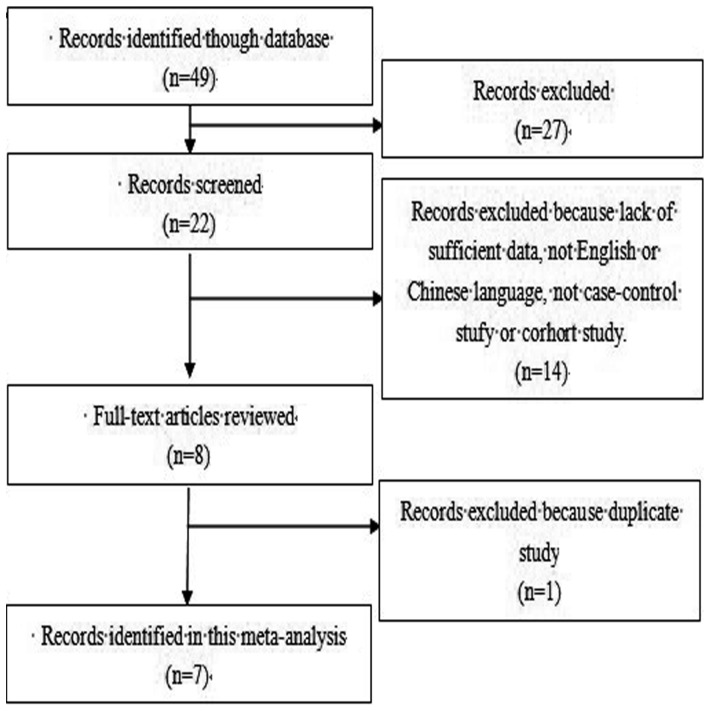
Flow chart demonstrating those studies that were processed for inclusion in the meta-analysis.

As showed in Figure 1, a total of seven studies were included in this meta-analysis including 3,213 cases and 3,849 controls [4]–[10]. The studies identified and their main characteristics were summarized in Table 1 and Table 2. Genotype distribution of six studies polymorphism did not differ from Hardy-Weinberg equilibrium with in control groups (all were greater than 0.05, Table 3).

**Table 2:** Characteristics of the Included Studies for Meta-analysis.

first author	publicationyear	location	ethnicity	Histology	study design	adjusted	Genotypingmethod	TP53BP1 polymorphism	cases(n)	controls(n)	Qualityscore
Frank B	2005	Germany	Caucasian	breast cancer	HB, CC	No	Taqman	D353E (rs560191),G412S (rs689647),K1136Q (rs2602141)	353	960	10
Ma H	2006	China	Asian	breast cancer	HB, CC	No	PCR	T-885G (rs1869258), Glu353 Asp(rs560191), Gln1136 Lys (rs2602141)	404	472	9
Chen K	2007	USA	Caucasian	squamous cellcarcinoma of thehead andneck	HB, CC	age,sex,ethnicity	PCR	T-885G (rs1869258), Glu353 Asp(rs560191), Gln1136 Lys (rs2602141)	818	821	10
Kiyohara C	2010	Japan	Asian	lung cancer	HB, CC	No	Taqman	Asp353 Glu (rs560191)	462	379	9
Naidu R	2011	Malaysia	Asian	breast cancer	HB, CC	age	PCR	T-885G (rs1869258), Glu353 Asp(rs560191)	387	252	9
Oliveira S	2012	Portugal	Caucasian	cervical cancer	HB, CC	No	Taqman	D353E (rs560191)	149	280	9
Zhang H	2013	China	Asian	lung cancer	HB, CC	gender,age,smoking status	Taqman	Glu353 Asp (rs560191), Gln1136 Lys(rs2602141),G412S (rs689647)	640	685	10

HB,hospital based; CC, case-comtrol; PCR, polymerase chain reaction.

**Table 3:** Distributions of TP53BP1 Genotype and Allele among Cases and Controls.

first author	study groups	Distribution of TP53BP1 genotypes			Frequency of TP53BP1 alleles		HWE for control
		AA	AG	GG	A alle	G alle	
Chen K	case	427	322	69	1176	460	0.45
	control	424	323	74	1171	471	0.27
Frank B	case	165	148	30	478	208	0.69
	control	453	405	94	1311	593	0.8
Kiyohara C	case	174	231	57	579	345	0.14
	control	110	188	81	408	350	0.96
Ma H	case	131	194	77	456	348	0.73
	control	144	237	85	525	407	0.46
Naidu R	case	160	189	38	509	265	0.09
	control	99	132	21	330	174	0.01
Oliveira S	case	21	63	65	105	193	0.36
	control	49	132	99	230	330	0.66
Zhang H	case	112	322	206	546	734	0.47
	control	144	338	203	626	744	0.88

HWE: Hardy-Weinberg equilibrium.

### Quantitative Data Synthesis

**Figure 2:**
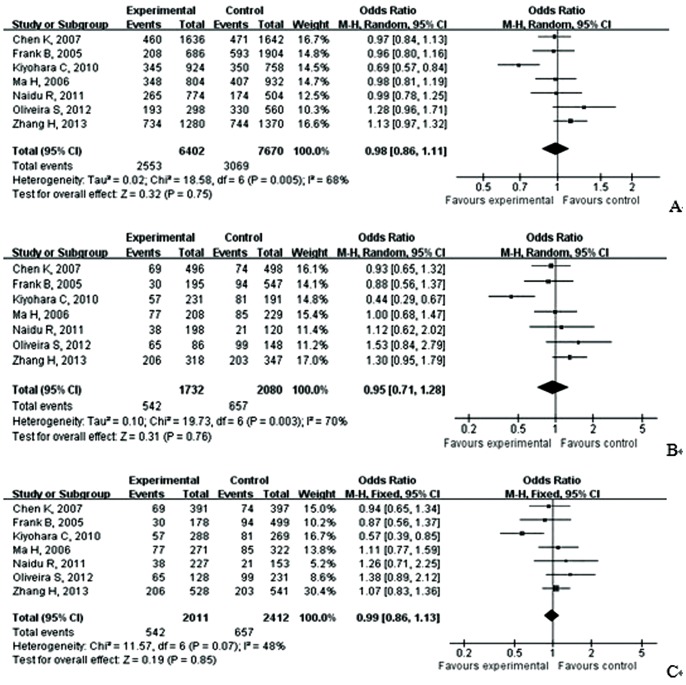
A. Forest plot of the association between cancer and the Glu353Asp (rs560191) mutation in overall population (G vs A). Figure 2.B. Forest plot of the association between cancer and the Glu353Asp (rs560191) mutation in overall population (GG vs AA). Figure 2.C. Forest plot of the association between cancer and the Glu353Asp (rs560191) mutation in overall population (GG vs AG).

**Figure 3:**
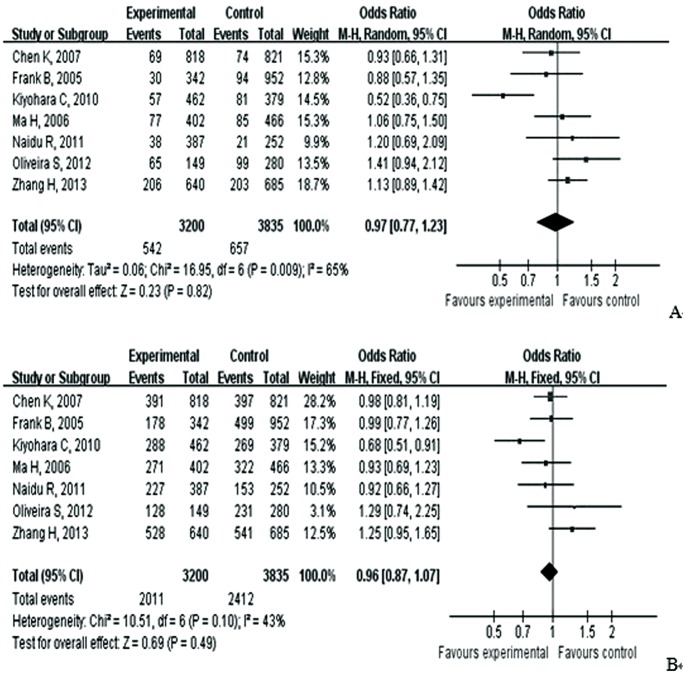
A. Forest plot of the association between cancer and the Glu353Asp (rs560191) mutation in overall population (GG vs AG+AA). Figure 3.B. Forest plot of the association between cancer and the Glu353Asp (rs560191) mutation in overall population (GG+AG vs AA).

**Figure 4:**
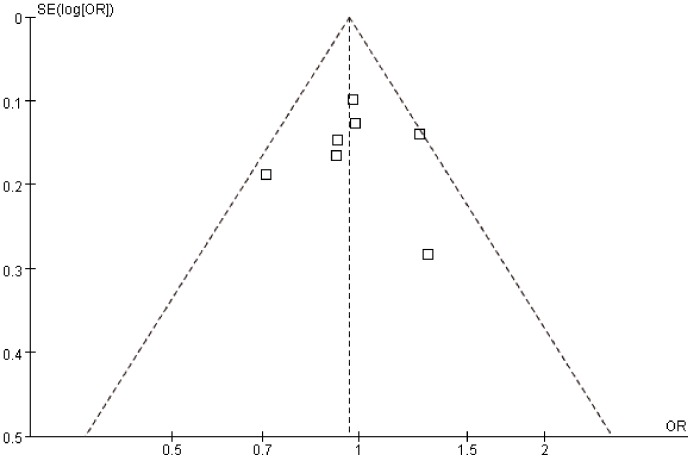
Funnel plot analysis on the detection of publication bias in the meta-analysis of the associations between Glu353Asp (rs560191) mutation and cancer risk.

As showed in Table 4, meta-analysis of the total studies showed that there was no association between Asp353Glu (rs560191) polymorphism and risk of cancer under all five genetic models in overall population (OR = 0.98, 95% CI = 0.86–1.11 for G versus A; OR = 0.95, 95% CI = 0.71–1.28 for GG versus AA; OR = 0.99, 95% CI = 0.86–1.13 for GG versus AG; OR = 0.97, 95% CI = 0.77–1.23 for recessive model; OR = 0.96; 95% CI = 0.87–1.07 for dominant model) (Figure 2 and Figure 3). In the subgroup analysis according to ethnicity, cancer type, adjusted with control or not, genotyping methods, HWE and quality score, the results suggested that Asp353Glu (rs560191) polymorphism were not associated with the risk of cancer. There was no significant publication bias according to Begg’s and Egger’s tests (Begg, p = 0.21; Egger, p = 0.64) and funnel plot (Figure 4).

**Table 4:** Summary ORs and 95% CI of the rs560191 Polymorphism in the TB53BP1 Gene and Cancer Risk.

Studygroups	Variables	AlleleModel				Codominantmodel								Recessivemodel				Dominantmodel			
		G vs. A(fixedmodel)			G vs. A(randommodel)	GG vs. AA(fixedmodel)			GG vs. AA(randommodel)	GG vs. AG(fixedmodel)			GG vs. AG(randommodel)	GG vs.AA+AG(fixedmodel)			GG vs.AA+AG(randommodel)	GG+AGvs. AA(fixedmodel)			GG+AGvs. AA(randommodel)
		OR(95% CI)	P_h_	I^2^%	OR(95% CI)	OR(95% CI)	P_h_	I^2^%	OR(95% CI)	OR(95% CI)	P_h_	I^2^%	OR(95% CI)	OR(95% CI)	P_h_	I^2^%		OR(95% CI)	P_h_	I^2^%	OR(95% CI)
Overall	7	0.98(0.91–1.05)	0.005	68	0.98(0.86–1.11)	0.96(0.82–1.12)	0.003	70	0.95(0.71–1.28)	0.99(0.86–1.13)	0.07	48	0.98(0.80–1.20)	0.99(0.86–1.12)	0.009	65	0.97(0.77–1.23)	0.96(0.87–1.07)	0.1	43	0.96(0.84–1.11)
Ethnicity																					
Caucasian	3	1.01(0.90–1.12)	0.22	34	1.02(0.89–1.18)	1.00(0.78–1.28)	0.29	19	1.01(0.76–1.34)	1.03(0.81–1.30)	0.29	20	1.03(0.79–1.35)	0.86(0.71–1.04)	0.004	77	0.88(0.58–1.31)	1.00(0.86–1.16)	0.64	0	1.00(0.86–1.16)
Asian	4	0.96(0.87–1.05)	0.002	80	0.94(0.76–1.16)	0.93(0.77–1.13)	0.0007	82	0.90(0.55–1.47)	0.97(0.81–1.15)	0.03	66	0.95(0.69–1.31)	0.96(0.82–1.13)	0.004	78	0.92(0.63–1.34)	0.93(0.80–1.08)	0.03	67	0.92(0.72–1.19)
Cancer type																					
Breast cancer	3	0.98(0.87–1.10)	0.98	0	0.98(0.87–1.10)	0.97(0.75–1.27)	0.8	0	0.98(0.75–1.27)	1.05(0.82–1.35)	0.57	0	1.05(0.82–1.36)	1.02(0.80–1.30)	0.66	0	1.02(0.80–1.30)	0.95(0.81–1.12)	0.92	0	0.95(0.81–1.12)
Lung cancer	2	0.94(0.83–1.06)	0.0001	93	0.89(0.55–1.43)	0.88(0.69–1.13)	<0.0001	94	0.77(0.27–2.21)	0.89(0.72–1.10)	0.008	86	0.80(0.43–1.46)	0.90(0.74–1.10)	0.0005	92	0.77(0.36–1.66)	0.94(0.77–1.14)	0.002	89	0.92(0.50–1.69)
Others	2	1.03(0.90–1.18)	0.1	63	1.08(0.83–1.41)	1.06(0.78–1.43)	0.16	50	1.12(0.69–1.81)	1.10(0.83–1.45)	0.18	44	1.11(0.76–1.62)	1.11(0.85–1.44)	0.12	58	1.13(0.75–1.70)	1.01(0.84–1.21)	0.35	0	1.01(0.84–1.21)
Study with matching																					
Yes	3	1.04(0.94–1.14)	0.35	4	1.04(0.94–1.15)	1.12(0.90–1.39)	0.36	1	1.12(0.90–1.40)	1.05(0.86–1.27)	0.67	0	1.05(0.86–1.27)	1.07(0.90–1.29)	0.61	0	1.07(0.90–1.29)	1.03(0.90–1.19)	0.25	27	1.04(0.87–1.23)
No	4	0.92(0.83–1.02)	0.003	78	0.94(0.75–1.18)	0.81(0.65–1.01)	0.004	78	0.86(0.53–1.39)	0.92(.76–1.13)	0.02	70	0.93(0.64–1.35)	0.90(0.74–1.09)	0.003	79	0.91(0.60–1.38)	0.89(0.77–1.04)	0.12	49	0.90(0.72–1.13)
Genotyping																					
PCR	3	0.98(0.88–1.09)	0.99	0	0.98(0.88–1.09)	0.98(0.77–1.25	0.86	0	0.98(0.77–1.25)	1.05(0.84–1.33)	0.65	0	1.05(0.83–1.33)	1.02(0.82–1.28)	0.72	0	1.02(0.82–1.28)	0.95(0.82–1.10)	0.92	0	0.95(0.82–1.10)
Taqman	4	0.98(0.89–1.08)	0.0003	84	0.98(0.77–1.26)	0.94(0.77–1.15)	0.0002	84	0.93(0.54–1.60)	0.95(0.801.13)	0.02	71	0.93(0.66–1.31)	0.97(0.82–1.14)	0.001	81	0.93(0.62–1.39)	0.98(0.84–1.13)	0.02	71	0.99(0.74–1.33)
HWE																					
Yes	9	0.98(0.91–1.05)	0.002	73	0.98(0.84–1.13)	0.95(0.81–1.11)	0.002	74	0.94(0.68–1.30)	0.97(0.84–1.12)	0.05	54	0.96(0.77–1.19)	0.97(0.85–1.12)	0.006	70	0.95(0.74–1.23)	0.97(0.87–1.08)	0.06	52	0.97(0.82–1.15)
No	1	0.99(0.78–1.25)	NA	NA	0.99(0.78–1.25)	1.12(0.62–2.02)	NA	NA	1.12(0.62–2.02)	1.26(0.71–2.25)	NA	NA	1.26(0.71–2.25)	1.20(0.96–2.09)	NA	NA	1.20(0.96–2.09)	0.92(0.66–1.27)	NA	NA	0.92(0.66–1.27)
Score																					
> = 10	3	1.03(0.93–1.13)	0.29	19	1.03(0.92–1.14)	1.06(0.86–1.31)	0.23	32	1.05(0.81–1.36)	0.99(0.83–1.20)	0.7	0	1.00(0.83–1.20)	1.03(0.86–1.22)	0.49	0	1.03(0.86–1.23)	1.04(0.91–1.19)	0.31	16	1.04(0.90–1.21)
<10	4	0.92(0.82–1.02)	0.003	78	0.95(0.75–1.21)	0.84(0.67–1.06)	0.003	79	0.91(0.53–1.55)	0.98(0.79–1.20)	0.01	72	1.01(0.67–1.52)	0.94(0.77–1.14)	0.002	80	0.97(0.61–1.53)	0.86(0.73–1.01)	0.17	41	0.88(0.70–1.10)

P_h_, P-value for test of heterogeneity; HWE: Hardy-Weinberg equilibrium; PCR, polymerase chain reaction; OR: odds ratio; CI: confidence interval.

### Sensitivity Analysis

According to sensitivity analysis, the results showed us that there was no substantial modification of our estimates after exclusion of individual studies, indicating that the results were stable (data not shown).

## Discussion

It is well known that SNPs may contribute to an individual’s susceptibility to cancer and TP53BP1 is a key component in the cellular response to DNA damage [19]. Therfore, the SNPs of TP53BP1 may play an important role in the etiology of cancer. The conclusion that TP53BP1 gene played an important role in DNA repair has been well-researched, but the functional relevance of TP53BP1 gene polymorphism has not been reported. It is possible that the sequence variation in the promoter and coding region of TP53BP1 might affect its transcription and downstream biological function [4], [5].

To the best of our knowledge, some researches that aim at the role of Asp353Glu (rs560191) polymorphism in cancer risk have been performed, but the results are controversial. In order to evaluate on the association between the Asp353Glu (rs560191) polymorphism and cancer risk, we performed this meta-analysis.

We have not found a sinificant association between TP53BP1 Asp353Glu (rs560191) polymorphism and cancer risk in overall population, but different ethnicity, study design, genotyping methods and cancer type would be responsible for the negtive conclusions. We perfomed subgroup analysis based on these factors. However, the resluts showed us that Asp353Glu (rs560191) polymorphism were not associated with the risk of cancer according to ethnicity, cancer type, study with matching or not, genotyping methods, HWE and study score. That may be because only one study [10] reported that the Asp353Glu polymorphism was associated with a risk of cancer. Therefore, further studies are needed to confirm our results.

Some studies indicate that TP53BP1 variants may have protective effects on squamous cell carcinoma of the head and neck (SCCHN) risk but such effects were confined to TP53 Arg72Pro variant allele/haplotype carriers [5], [8]. As the reason for few studies were perfomed and there were many meta-analysis related on TP53 Arg72Pro polymorphism and cancer risk [20], [21], we could not use meta-analysis to analyze the relationship between TP53BP1 Asp353Glu (rs560191) polymorphism combined with TP53 gene polymorphism and cancer. In addition, Rudd et al. [22] and Truong et al. [23] found that Asp353Glu (rs560191) polymorphism was associated with lung cancer risk, but this association was not been found in the study [24] by Brooks JD et al. In addition, because lack of sufficient data from these three studies, we could not include these studies in this meta-analysis. That may be another reason for the negtive conclusion in this meta-analysis.

The meta-analysis by Timofeeva et al. [25] did not show a significant association between rs560191 polymorphism and lung cancer risk. It came to the same conclusion with our study. However, it was only concerned lung cancer risk. In our meta-analysis, the association between rs560191 polymorphism and other cancer types including cervical cancer, breast cancer and squamous cell carcinoma of the head and neck was also analyzed.

There are several limitations in this meta-analysis that should be considered. First, cancer is a multi-factorial disease including complex interactions from environmental exposure to gene factors. In this meta-analysis, we had insufficient data to perform an evaluation of such interactions for the independent role of TP53BP1 Asp353Glu (rs560191) polymorphism in cancer development. Second, only seven studies were included in this meta-analysis. Thus, more studies are needed to identify this association more comprehensively. Third, study by Naidu et al. [4] showing genotype distributions of the control population that were not in HWE was included in this meta-analysis. Forth, we did not consider studies published in languages other than English/Chinese or data presented in abstracted form; thus, publication and potential language biases may occur.

In conclusion, this meta-analysis suggested that the polymorphism in TP53BP1 Asp353Glu (rs560191) gene could not be regarded as a genetic risk factor for cancer. At the same time, this result should be interpreted cautiously. To verify this result, large scale case-control studies with detailed individual information are needed.

## Supporting Information

Checklist S1 **PRISMA 2009 Checklist.**
(DOC)
